# A new approach: Laparoscopic right hemicolectomy with priority access to small bowel mesentery

**DOI:** 10.3389/fsurg.2022.1064377

**Published:** 2023-01-05

**Authors:** Feng Pi, Xudong Peng, Chaozheng Xie, Gang Tang, Yuhao Qiu, Zhenzhou Chen, Zhengqiang Wei

**Affiliations:** Department of Gastrointestinal Surgery, The First Affiliated Hospital of Chongqing Medical University, Chongqing, China

**Keywords:** laparoscopy, colon tumor, right hemicolectomy, surgery, priority access to the small bowel mesentery

## Abstract

**Background:**

For laparoscopic right hemicolectomy, the intermediate approach is commonly employed. However, this approach possesses several disadvantages. In this study, we compare priority access to the small bowel mesentery and the intermediate approach.

**Methods:**

The clinical data of 196 patients admitted to the First Hospital of Chongqing Medical University for laparoscopic right hemicolectomy from January 2019 to January 2022 were retrospectively collected and divided into the small bowel mesenteric priority access and traditional intermediate access groups. The operative time, intraoperative bleeding, number of lymph node dissection, postoperative anal venting time, toleration of solid and liquid intake, and postoperative hospital stay and complications were compared between the two different approaches.

**Results:**

In total, 81 cases of small bowel mesenteric priority access and 115 cases of intermediate approach for right hemi-colonic radical resection were compared. The operative time was 191.98 ± 46.05 and 209.48 ± 46.08 min in the small bowel mesenteric priority access and intermediate access groups, respectively; the difference was statistically significant. There were no significant differences in the intraoperative bleeding and lymph node clearance. However, the scatter plot analysis showed that severe intraoperative bleeding was relatively less frequent in the small mesenteric priority access group, compared with that in the intermediate approach group. Additionally, there were no statistically significant differences in the first exhaust and defecation times, hospital stay after operation, toleration of solid and liquid intake, and postoperative complication between the two groups.

**Conclusion:**

In laparoscopic right hemicolectomy, the small bowel mesenteric priority approach can significantly shorten the operation time compared with the intermediate approach. It can reduce intraoperative bleeding and the operation is simple and safe to perform, making it suitable for less experienced surgeons. Therefore, the small bowel mesenteric priority approach has the potential to be a suitable alternative and deserves further clinical promotion and application.

## Introduction

Colorectal cancer is a major disease threatening human health (1–3), with its diagnosis and treatment arousing immense concern worldwide (4–6). Laparoscopic radical colorectal resection has become an established technique for treating colorectal cancer. It includes complete mesocolic excision (CME) and total mesocolic excision (TME) (7–9), which are two surgical methods that have become gradually standardized in the recent years. However, the choice of access for laparoscopic radical colorectal resection remains controversial.

Compared with laparoscopic radical rectal cancer resection, laparoscopic radical right hemicolectomy is more complicated, and the surgical approach and criteria for its selection has undergone many changes, owing to factors such as vascular variation, difficulties in locating vital, adjacent tissues and organs and performing colon lymph node dissection (10, 11). Among the possible surgical approaches, the “intermediate approach” proposes ligation of the dissociated mesenteric vessels first, and better aligns with the principle of the no touch isolation technique in surgical oncology (12–14). Due to the aforementioned difficulties, surgeons experienced with laparoscopic techniques often prefer the intermediate approach when performing laparoscopic radical right hemicolectomy. However, the disadvantage of the intermediate approach is that the complexity of colon anatomy may lead to difficulties in performing operation steps (15, 16), such as those involving separating and revealing mesenteric-related vessels in obese patients, accessing the correct anatomical plane, and disconnecting the vessels in areas where lesions are located. Before it become clear whether a tumor can be radically resected, the operator may be very passive.

**Figure 1:**
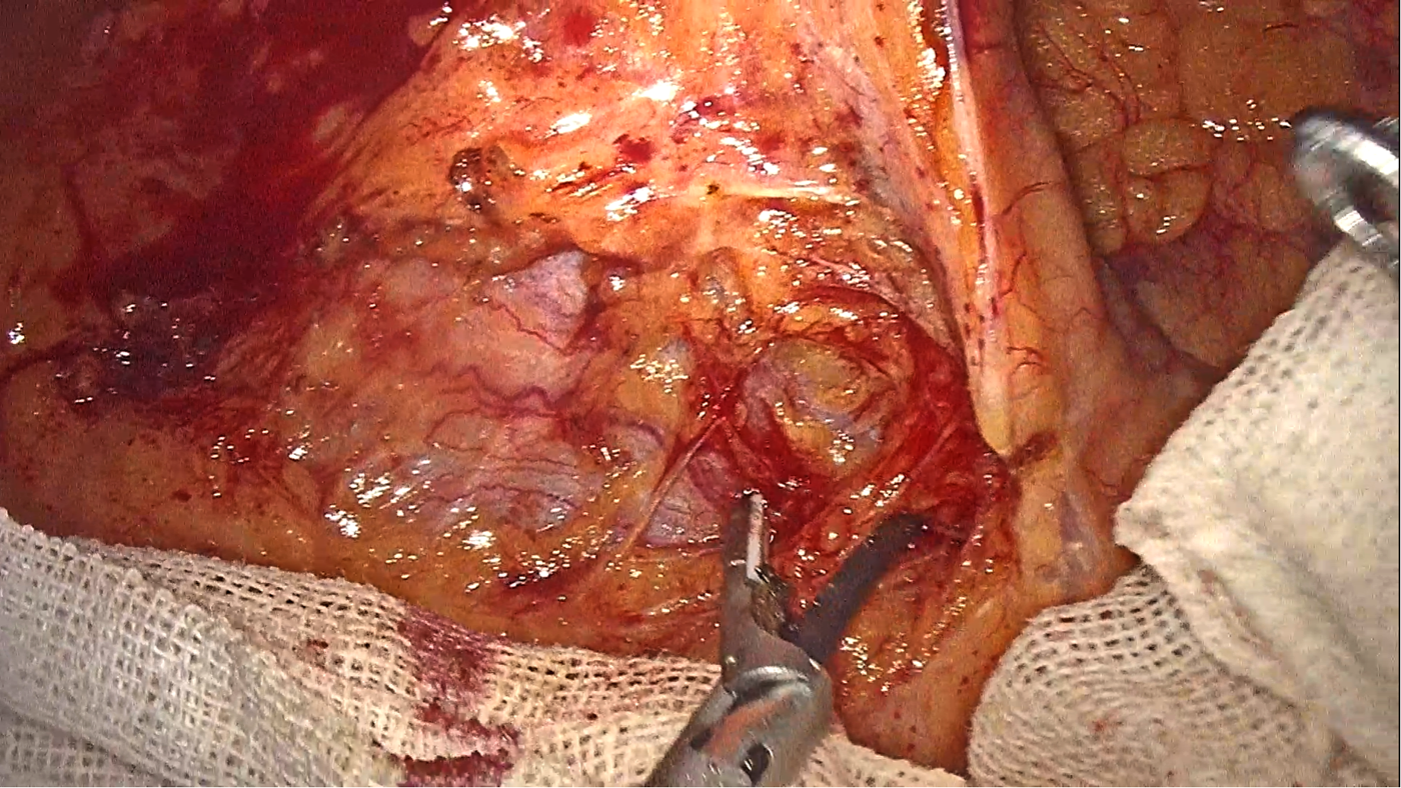
Toldt gap cannot be found in the intermediate approach (narrow field of view, no obvious reference or gap in the field, indistinguishable tissue structures below the open level, bleeding makes the gap more difficult to find).

Additionally, the risk of injury to both Henle's stem and superior mesenteric vessels is increased due to large anatomical variations after right hemicolectomy (Figure 1). The transition from the duodenum to the pancreas head surface is also prone to damage by inadvertent entry into the pancreatic tissue, resulting in bleeding (17).

Finally, the approach is challenging for novice surgeons to perform; thus, surgeons are constantly exploring new surgical approaches and improved points of access (18, 19). In this study, we compared the clinical data from patients who underwent the small bowel mesenteric priority approach and the traditional intermediate approach for radical right hemicolectomy.

## Methods

### Search strategy

A total of 196 patients were admitted to the First Hospital of Chongqing Medical University for laparoscopic radical right hemicolectomy to treat right colon cancer which from January 2019 to January 2022. Each procedure was performed by an experienced chief surgeon. Their clinical data were retrospectively collected and included in this study. Patients were divided into two groups: those who underwent the small bowel mesenteric priority approach and those who underwent the traditional intermediate approach.

### Surgical approach

Patients from the intermediate approach group were placed in the supine position, and a 5-hole Trocar puncture was used to turn the greater omentum towards the liver and stomach. The small intestine was moved to the left upper abdomen, with the ileocecal region as a guide to fully reveal the “yellow-white junction line” between the root of the ileocecal mesentery and the retroperitoneum, and the right Toldt space was entered and extended to the right and cephalic side. The left edge of the superior mesenteric artery was treated with the ileocolic, right and midcolic vessels for D3 lymph node dissection, after which the gastrocolic and hepato colic ligaments were severed and the right colon was freed. Finally, the specimen was removed through a median epigastric incision and intestinal resection anastomosis was performed (20).

Patients from the small bowel mesenteric access group had a 10-mm Trocar inserted in the lower left umbilicus, a 12-mm Trocar inserted in the lower left umbilicus, a 5-mm Trocar inserted at the Mai's point, and a 5 mm Trocar placed under the rib arch in the left abdomen and left midclavicular line. Intraoperative separation was first performed laparoscopically. The transverse colonic mesentery was lifted by a surgical assistant to reveal the direction of mesenteric vascular alignment. Then, the small intestine was dissected along the inferior edge of the ileocolic vessels at the anticipated separation of the small intestine 10–15 cm from the ileocecal region, and the corresponding segment of the small intestine was naked. After clearing the lymphatic adipose tissue at the root of the vessel, the colonic branch of the gastrocolic trunk vessel was cut off with a hemolock clamp, and the ascending colon and ileocecal part were separated from the inner side. The transverse colon was lifted upward to reveal the transverse colonic mesentery. After recognizing the mesocolic vessels, the roots of the mesocolic vessels and the lymphatic adipose tissue along them were cleared and the right branch of the mesocolic vessels was cut off with hemolock clamps. Subsequently, the gastrocolic ligament was lifted and separated to the right to the hepatic flexure of the colon with an ultrasonic knife followed by the release of the hepatic flexure adhesions. The right half of the transverse colon was freed, and the lateral peritoneum was excised from the lateral part of the ileocecal and ascending colon. The greater omentum was cut in the middle, and the gastrocolic ligament was separated from the pre-excision site. Then, the transverse colon was separated from the pre-excision site by nudging.

**Figure 2:**
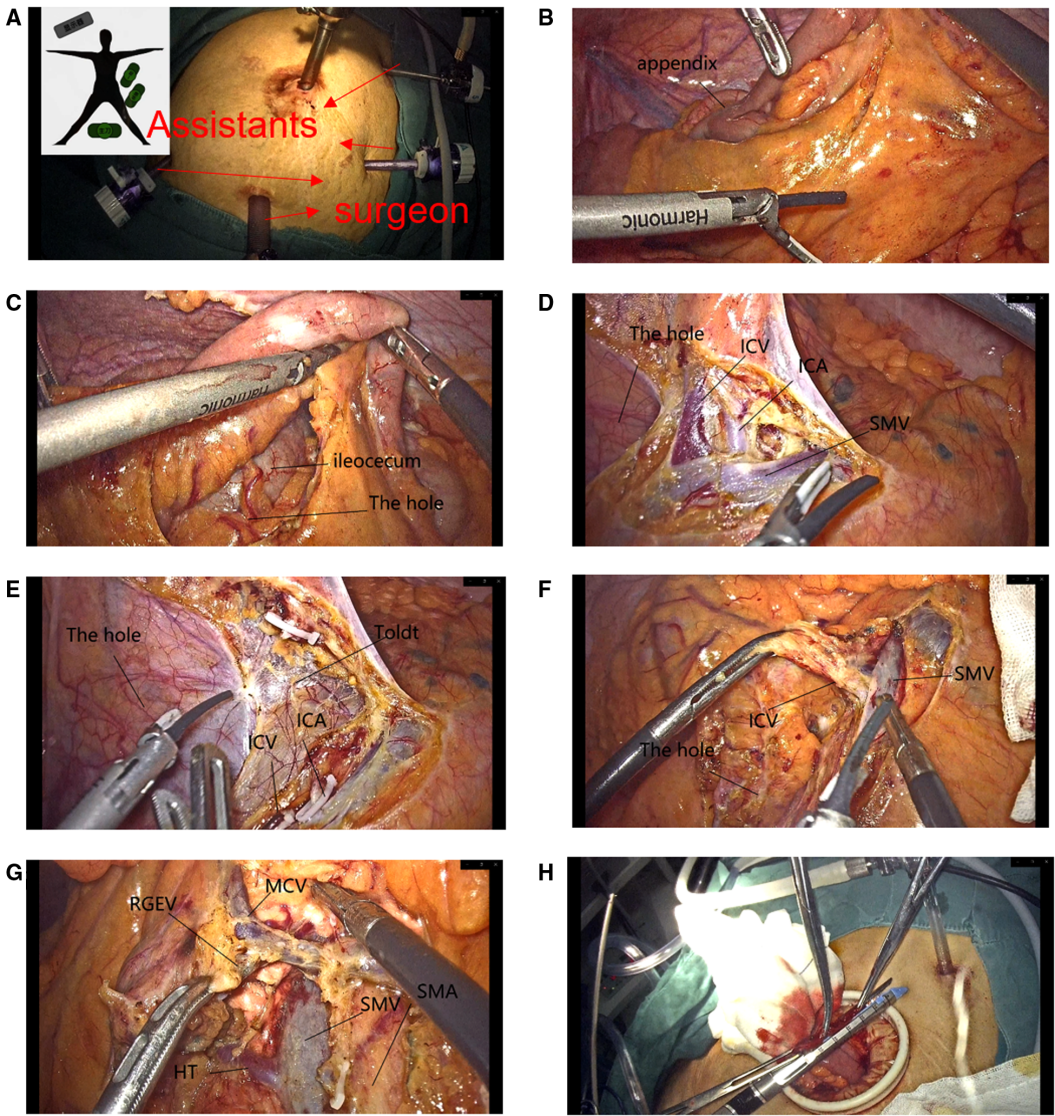
(**A**) Trocar. (**B**) Opened directly through the mesentery of the small intestine to create a “hole”. (**C**) Enlargement of the “hole” allows clear visualization of the inferior mesenteric structures of the small intestine and ileocecum. (**D**) The superior mesenteric vein along the ileocolic vein is easy to find. (**E**) The gap was extended to the ascending colon and the ileocecal region by cutting the blood vessels while extending the gap. (**F**) Dissection of the ileocolic vessels. (**G**) Overall vascular. (**H**) Anastomosis of the small intestine and transverse colon.

A 5-cm median parasternal incision was made in the abdomen, and the ileum was cut at the preexcised site of the naked small intestine. Finally, the specimen was removed and ileo-transverse colonic anastomosis was performed (Figure 2).

### Study selection

The inclusion criteria were as follows: the tumor was in the right colon; colon cancer was clearly identified by preoperative colonoscopy and pathological examination; the tumor was confined to the intestinal wall and did not invade the posterior peritoneum and surrounding organs; and patients underwent extracorporeal anastomosis.

### Data extraction

The exclusion criteria were as follows: extensive tumor infiltration; distant metastasis such as that in the liver and lung; intestinal obstruction; a history of abdominal surgery, extensive adhesions in the abdominal cavity, and the inability to perform laparoscopic surgery for exploration; patients did not undergo extracorporeal anastomosis.

### Statistical analysis

The count data were expressed as percentages (%) and chi-squared (*x*^2^) tests, while the measurement data were expressed as means ± standard deviations (*x* ± *s*). For comparison of the two groups and multiple groups, the t-test and one-way ANOVA were conducted, respectively. Data analysis was conducted using SPSS 22.0 (IBM, Armonk, NY, USA). A statistical value of *P* < 0.05 indicated statistical significance.

## Results

### Baseline data

In this study, there were 81 cases of small bowel mesenteric priority access and 115 cases of traditional laparoscopic right hemi-colonic radical resection. There were no statistically significant differences in the demographics data of patients, including sex, age, tumor site and tumor stage etc. between the two groups (Table 1).

**Table 1:** Comparison of preoperative baseline data between the two groups of patients (*n* = 196).

Characteristics	1^a^ (*N* = 81)	2^b^ (*N* = 115)	Total (*N* = 196)	*P* value
Year	61.09 ± 13.95	63.23 ± 13.26	62.34 ± 13.55	0.28
BMI	22.78 ± 2.61	22.16 ± 3.48	22.41 ± 3.16	0.18
Total protein	67.68 ± 7.60	65.93 ± 7.48	66.80 ± 7.54	0.11
Albumin	39.02 ± 5.64	38.90 ± 5.14	38.96 ± 5.33	0.88
Hemoglobin	107.08 ± 29.56	107.24 ± 26.43	107.16 ± 27.63	0.97
Charlson comorbidity index	5.26 ± 1.97	5.53 ± 1.74	5.40 ± 1.87	0.30
Gender				0.67
Female	41 (20.92%)	63 (32.14%)	104 (53.06%)	
Male	40 (20.41%)	52 (26.53%)	92 (46.94%)	
Stage				0.44
1	11 (5.61%)	12 (6.12%)	23 (11.73%)	
2	37 (18.88%)	63 (32.14%)	100 (51.02%)	
3	33 (16.84%)	40 (20.41%)	73 (37.24%)	
Anatomical_location				0.86
Ascending colon	33 (16.84%)	45 (22.96%)	78 (39.80%)	
Hepatic flexure	29 (14.80%)	39 (19.90%)	68 (34.69%)	
Ileocecal	19 (9.69%)	31 (15.82%)	50 (25.51%)	
Hypertension				0.42
0	54 (27.55%)	84 (42.86%)	138 (70.41%)	
1	27 (13.78%)	31 (15.82%)	58 (29.59%)	
Diabetes				0.11
0	66 (33.67%)	104 (53.06%)	170 (86.73%)	
1	15 (7.65%)	11 (5.61%)	26 (13.27%)	

^a^ The small bowel mesenteric priority approach.

^b^ The traditional intermediate approach.

### Comparison of intraoperative conditions

**Figure 3:**
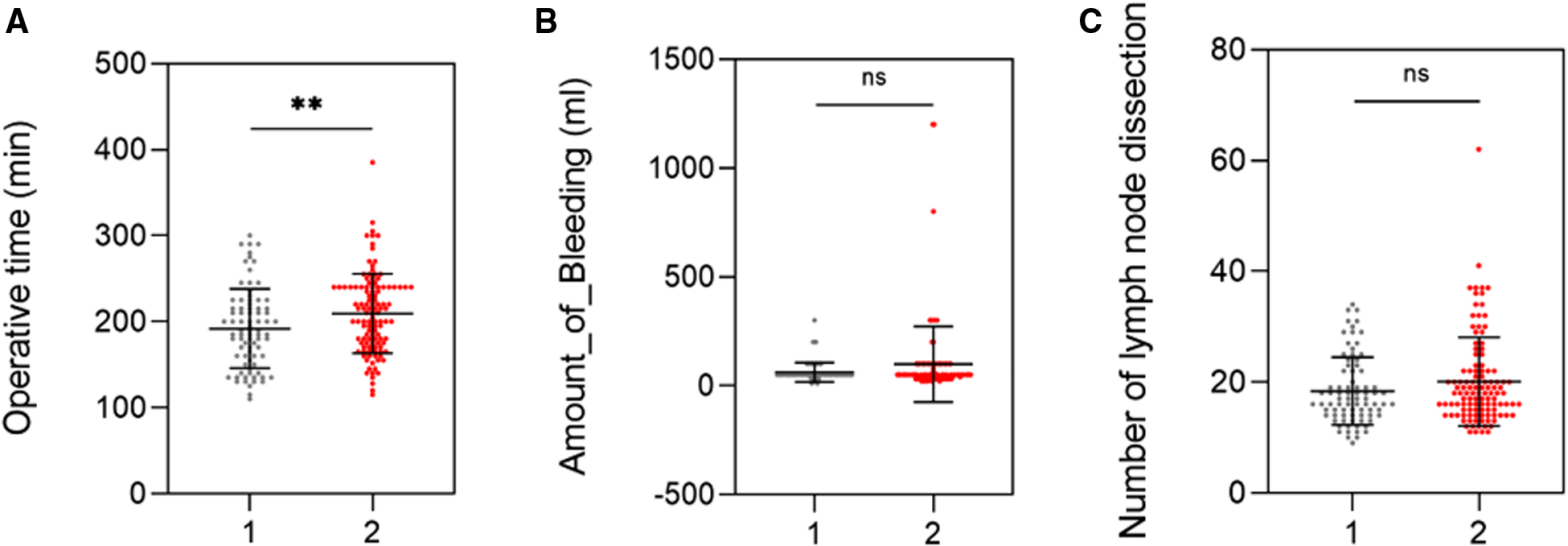
Comparison of intraoperative variables between the two groups of patients. (**A**) Operative time. (**B**) Amount of bleeding. (**C**) Number of lymph node dissection.

Intraoperative conditions were compared between the small bowel mesenteric priority approach and the traditional intermediate approach for radical right hemicolectomy for colon cancer (Figure 3). The results showed that the operative time was shorter in the small mesenteric priority access group (191.98 ± 46.05) than that in the conventional intermediate access group (209.48 ± 46.08) (Figure 3A). Furthermore, although the difference in intraoperative bleeding was not statistically significant (Figure 3B), scatter plot analysis showed relatively fewer cases of high intraoperative bleeding in the small bowel mesenteric priority access group, compared with that in the intermediate approach group. The highest intraoperative bleeding volume was 300 ml. In addition, the number of lymph nodes cleared was similar for both approaches (18.38 ± 6.11 vs. 20.10 ± 7.98) (Figure 3C) and was not statistically significant (The former data is the small bowel mesenteric priority approach, and the latter is the traditional intermediate approach.). However, these results strongly suggest the feasibility of the small bowel mesenteric priority approach.

### Comparison of postoperative recovery

The time to first anal discharge and bowel movement was 2.96 ± 0.80 vs. 3.09 ± 0.97 days and 3.95 ± 1.16 vs. 3.89 ± 1.26 days, in the small bowel mesenteric priority approach and traditional intermediate approach groups, respectively. Similarly, the time to tolerate fluid and semi-fluid was 2.65 ± 1.70 vs. 2.78 ± 1.39 and 6.44 ± 3.63 vs. 6.21 ± 1.98 days (The former data is the small bowel mesenteric priority approach, and the latter is the traditional intermediate approach.), in the small bowel mesenteric priority approach and traditional intermediate approach groups, respectively (Table 2).

**Table 2:** Comparison of postoperative indexes between two groups of patients (*n* = 196, *x* ± *s*).

Characteristics	1^a^ (*N* = 81)	2^b^ (*N* = 115)	Total (*N* = 196)	*P* value
First_exhaust_time				0.34
Mean ± SD	2.96 ± 0.80	3.09 ± 0.97	3.04 ± 0.90	
First_defecation_time				0.72
Mean ± SD	3.95 ± 1.16	3.89 ± 1.26	3.91 ± 1.22	
Time_of_first_tolerance_to_liquid				0.56
Mean ± SD	2.65 ± 1.70	2.78 ± 1.39	2.73 ± 1.52	
Time_of_first_solid_tolerance				0.56
Mean ± SD	6.44 ± 3.63	6.21 ± 1.98	6.31 ± 2.78	
Hospital_days_after_operation				0.97
Mean ± SD	8.36 ± 4.10	8.37 ± 2.30	8.37 ± 3.16	
Ileus	2	6	8	
Anastomotic leak	0	1	1	
Incision infection	0	1	1	
Lung infection	2	11	13	
Abdominal infection	17	19	36	
Chylous ascites	7	4	11	
Anastomotic stenosis	1	0	1	
Postoperative_complication				0.91
0	57 (29.08%)	83 (42.35%)	140 (71.43%)	
1	24 (12.24%)	32 (16.33%)	56 (28.57%)	

^a^ The small bowel mesenteric priority approach.

^b^ The traditional intermediate approach.

There were no postoperative deaths in both groups; the incidence of postoperative complications was 12.24% and 16.33% in the two groups, respectively, with no statistically significant difference. There were 19 cases of abdominal infection, 4 cases of chylous ascites, 11 cases of pulmonary infection, 6 cases of intestinal obstruction, 1 case of incisional infection, 0 cases of anastomotic stenosis and 1 case of anastomotic leakage in the intermediate access group, in addition to 17 cases of abdominal infection, 7 cases of chylous ascites, 3 cases of pulmonary infection, 2 cases of intestinal obstruction, 0 cases of incisional infection, 1 case of anastomotic stenosis and 0 cases of anastomotic leakage in the small bowel mesenteric priority access group.

## Discussion

Right colon cancer includes malignant tumors occurring in the cecum, ascending colon, and hepatic flexure. With the popularization and development of laparoscopic surgery, laparoscopic D3 lymph node dissection and CME for radical right hemicolectomy has become the standard for right colon cancer treatment (21) due to many procedural advantages, such as less trauma, accurate access at an anatomical level, standardized ligation of the mesenteric root vessels and lymph node dissection, and fewer complications and shorter recovery time for patients (22, 23). However, radical resection of right hemicolectomy includes its drawbacks, particularly vascular variation and difficulty in removing colonic lymph nodes. Furthermore, it involves several important organs and tissues such as the ureter, pancreas, and duodenum (24, 25), hence that the selection of an appropriate approach is vital to surgery success.

The traditional intermediate approach is currently the most widely used approach. However, this approach has many disadvantages. First, when searching for the Toldt gap, the mesentery must to be incised from below the ileocolic vessels. Due to the large vascular variation, bleeding during the separation of the vessels leads to an unclear field, and it is easy to enter the wrong level which results, in bleeding and injury to the ureteral genital vessels and retroperitoneal organs such as the anterior renal fascia. Furthermore, the integrity of the colonic mesentery is damaged. The anatomy of the middle approach from the duodenum to the anterior space of the pancreatic head may cause accidental injury the stem of Henle and entry into the pancreatic tissue, resulting in hemorrhage and unclear vision. Another disadvantage is that in obese patients, as it is challenging to enter the correct level through the middle approach in these patients. As the anatomical layers of obese patients are not well demarcated, they can lead to bleeding and complications during the operation (26). Finally, the complexity of the operation of the intermediate approach creates a steep learning curve for novice surgeons, thus requiring both experienced surgeons and surgical assistants.

This study retrospectively analyzed the clinical data of patients undergoing laparoscopic radical right hemicolectomy for right colon cancer in our gastrointestinal surgery department from 2019 to 2022. The clinical results were compared between patients who underwent the small bowel mesenteric priority approach and those who underwent the traditional intermediate approach for radical right hemicolectomy. The results showed that the operation time of the small bowel mesenteric priority approach (experimental group) was shorter than that of the traditional intermediate approach (control group). Although the difference in intraoperative bleeding was not statistically significant, the scatter plot analysis showed that the priority small bowel mesenteric approach reduced the risk of intraoperative hemorrhage, compared with that of the intermediate approach. The two approaches were consistent in terms of the number of lymph nodes cleared and postoperative recovery.

The feasibility and favorable clinical results of the priority access to the small intestine mesentery are well demonstrated. This may be because when compared with the traditional intermediate approach, the small intestine mesenteric approach has several advantages. The small intestine mesentery at the expected dissection point is cut first, which is conducive to judging the blood supply at the dissected small intestine after completion of the right hemicolectomy, thus preventing the occurrence of anastomotic leak due to the loss of blood supply at the anastomosis. Additionally, because the anatomical position of the ileocolic vein is relatively fixed, the first large vessel revealed after vertically cutting the small intestinal mesentery is the ileocolic vein. This avoids the difficulty in determining the location and course of the right hemi-colon vessels and reduces bleeding caused by accidental damage to vessels and tissues during surgery. Another advantage of the small intestine mesenteric priority approach is that it opens the small intestine mesentery first, which prepares for the subsequent step with the genetic branches of the mesenteric vessels. It also clears the operation field as the Toldt gap is expanded while dissecting the vessels. Furthermore, the small intestine mesentery is opened first, so the operator can cut the small intestine mesentery and can extend the Toldt gap to the caudal side by cutting the “hole” in the small intestine mesentery. This approach also opens the medial gap at the largest level, which is more convenient for the implementation of total colonic mesocolic resection. In this study, the surgical operations in both the control and experimental groups were performed by an experienced surgeon, and the learning curve for this surgeon who was proficient in the traditional method (25 cases) and on the learning curve for the new method (13 cases). The new method has been widely promoted and studied among young surgeons in our hospital, and for surgeons who mastered laparoscopic operations and were proficient in gastrointestinal anatomy, the learning curve was concentrated in 10–15 cases The learning curve for the traditional method in our hospital is 25–30 cases, which is also more aligned with the global learning curve markers (27, 28). Therefore, it can also be concluded that the new method is more simple for novices to learn and master. However, due to the small number of young surgeons in our hospital, CUSUM analysis has not been used for the time being, and the accurate learning curve of the new method needs to be further analyzed.

The approach avoids the need to turn the intestinal canal to find the caudal approach gap to expand the right colonic gap, reducing both operation time and patient discomfort, especially for patients who have obesity and mesenteric hypertrophy. It also reduces the risk of damaging blood vessels on the surface of Henle's stem and the pancreatic head. Even if there are accidental injuries to the vessels, it will be easier to handle due to greater exposure.

This study has some limitations. First, it is limited to the intermediate approach and the small bowel mesenteric priority approach. Future studies can explore the comparison of the therapeutic efficacy of the small bowel mesenteric priority approach with other approaches or combined approaches, and the results may improve the precision of laparoscopic techniques and clinical outcomes. Second, this is a single-center, retrospective study with a relatively small sample size and a limited study duration. It is necessary to validate the results of this study through conducting future studies with longer duration and large sample sizes.

In conclusion, compared with the traditional intermediate approach, the small bowel mesenteric priority approach in laparoscopic right hemicolectomy has the advantages of safety, minimal invasiveness, simplicity, and good operability. It is more conducive to ensuring an adequate surgical field and accurate anatomical positioning, and it can ensure similar clinical treatment results while better reducing the operating time, intraoperative bleeding, and other indicators.

## Data Availability

The original contributions presented in the study are included in the article/**Supplementary Material**, further inquiries can be directed to the corresponding author.
