# Supplementary figures and images for: A new approach: Laparoscopic right hemicolectomy with priority access to small bowel mesentery
Source: Front Surg. 2023 Jan 5;9:1064377. doi: 10.3389/fsurg.2022.1064377 (PMC9849593; doi:10.3389/fsurg.2022.1064377)

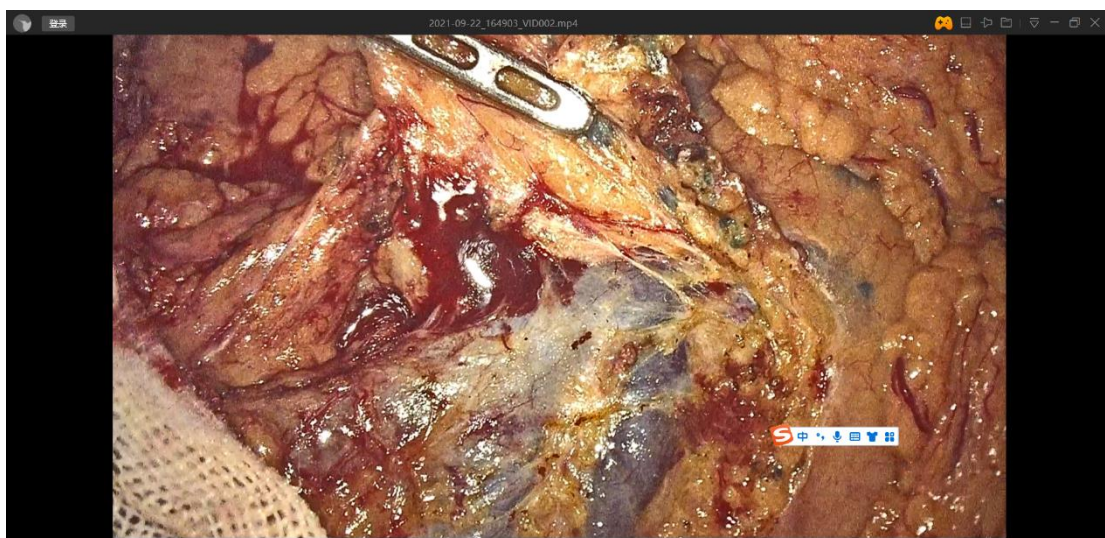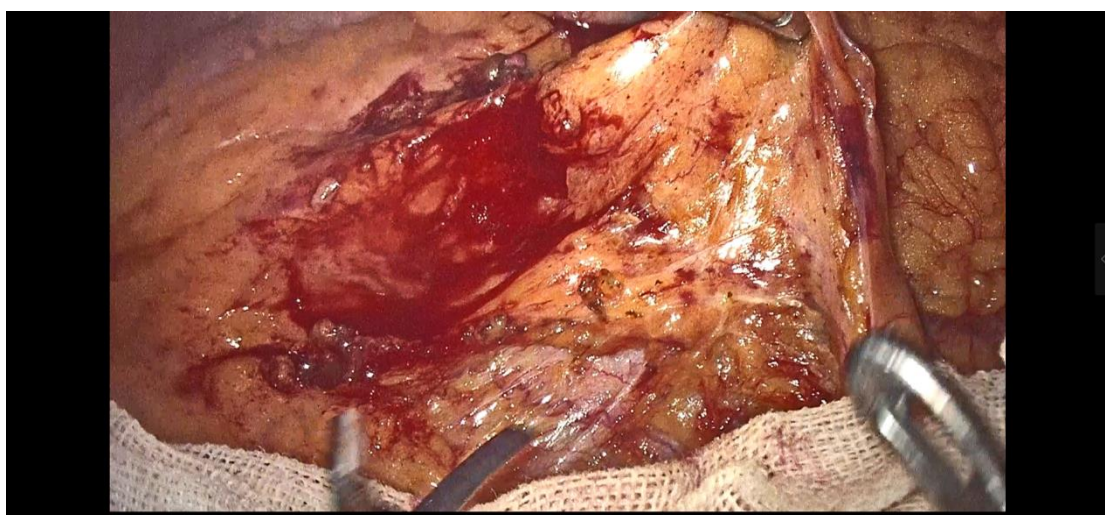

Supplement: Supplementary file 2 [file Datasheet1.pdf]
